# Recent Progress in Bio-Responsive Drug Delivery Systems for Tumor Therapy

**DOI:** 10.3389/fbioe.2022.916952

**Published:** 2022-06-29

**Authors:** Xiufeng Cong, Jun Chen, Ran Xu

**Affiliations:** ^1^ Department of Oncology, Shengjing Hospital of China Medical University, Shenyang, China; ^2^ Department of Thoracic Surgery, Shengjing Hospital of China Medical University, Shenyang, China

**Keywords:** tumor microenvironment, bio-responsiveness, cancer therapy, drug delivery system, pathophysiological characteristics

## Abstract

Spatially- and/or temporally-controlled drug release has always been the pursuit of drug delivery systems (DDSs) to achieve the ideal therapeutic effect. The abnormal pathophysiological characteristics of the tumor microenvironment, including acidosis, overexpression of special enzymes, hypoxia, and high levels of ROS, GSH, and ATP, offer the possibility for the design of stimulus-responsive DDSs for controlled drug release to realize more efficient drug delivery and anti-tumor activity. With the help of these stimulus signals, responsive DDSs can realize controlled drug release more precisely within the local tumor site and decrease the injected dose and systemic toxicity. This review first describes the major pathophysiological characteristics of the tumor microenvironment, and highlights the recent cutting-edge advances in DDSs responding to the tumor pathophysiological environment for cancer therapy. Finally, the challenges and future directions of bio-responsive DDSs are discussed.

## 1 Introduction

With the aggravation of population aging and the increase in life stress, the incidence and death rate of malignant tumors is increasing (Berben et al., 2021). According to the results from Global Cancer Statistics 2018 (Bray et al., 2018), there are approximately 18.1 million new cancer cases and approximately 9.6 million deaths due to cancer worldwide. In clinical practice, conventional cancer therapeutic strategies mainly include surgery, radiotherapy, chemotherapy, and immunotherapy (Lissoni et al., 2009). However, some patients lose the opportunity for surgery when they are diagnosed at a later stage (Wang et al., 2013). Additionally, considering the application limitation of radiotherapy and immunotherapy including tumor types, response rates, and high cost, traditional chemotherapy still plays an irreplaceable role in cancer therapy and will not be obsolete any time soon (Baxevanos and Mountzios, 2018; Sadeghi Rad et al., 2021; Fang, 2022). However, due to their low tumor accumulation, multidrug resistance, and lack of tumor selectivity, cytotoxic chemotherapy drugs and molecular targeted drugs commonly used in clinical practice often have serious side effects for normal tissues, which is one of the main formidable challenges faced by clinical chemotherapy (Shi et al., 2017; Hejmady et al., 2020). Currently, the application of drug delivery systems (DDSs), such as polymers, liposomes, dendrimers, biomolecular nanoparticles, and inorganic nanoparticles, has become a hot research topic in the field of chemotherapy because of their advantages in pharmacokinetics, targeting accumulation, high therapeutic efficiency, and lower organ toxicity (Ensign et al., 2014; Navya et al., 2019). Although DDS have many advantages compared to free drugs, the therapeutic effect remains unsatisfactory due to the heterogeneity of the EPR effect (Fang et al., 2020; Islam et al., 2021). For example, Doxil, the first FDA-approved nanomedicine, showed decreased cardiotoxicity in clinical use, but the overall clinical prognosis of patients with cancer treated with Doxil was not significantly improved (Barenholz, 2012). To address this dilemma, researchers have focused their attention on the design of various responsive DDSs according to different stimulus signals in the tumor microenvironment to realize rapid intratumoral or intracellular drug release and enhance tumor cell killing efficacy (Fang et al., 2011; Dou et al., 2020).

**SCHEME 1:**
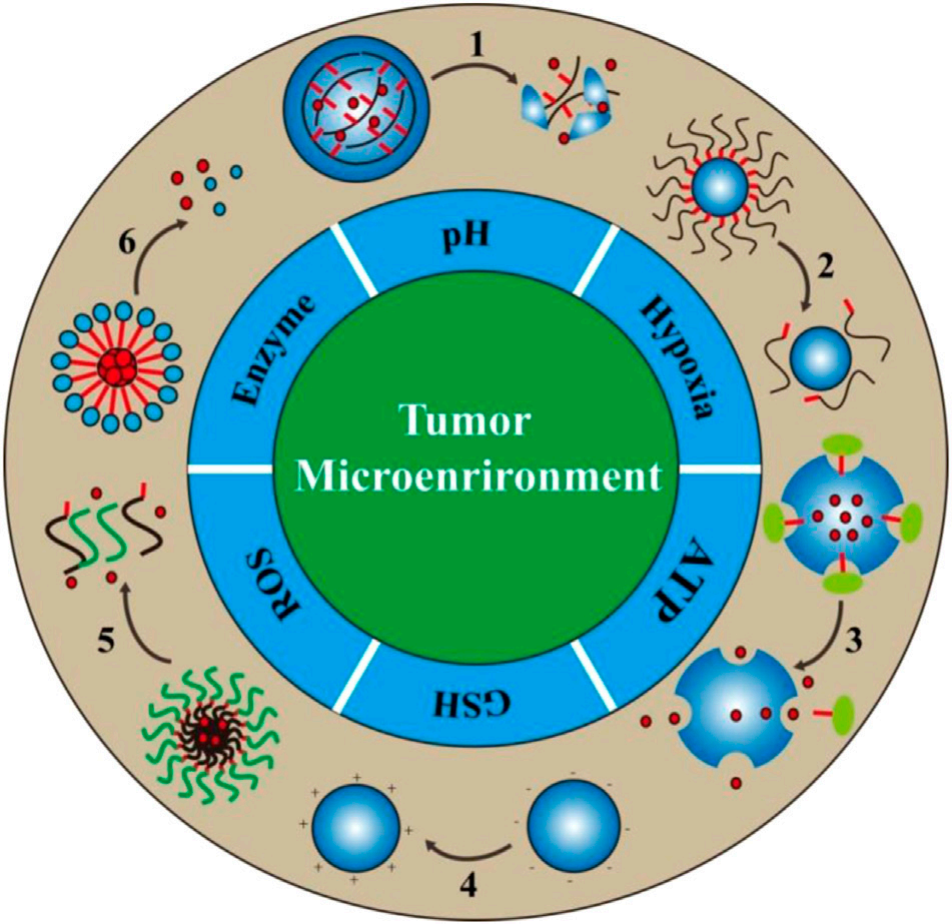
Schematic illustration of the design/action mechanism of responsive DDSs based on the abnormal pathophysiological characteristics in the tumor microenvironment. There are six major tumor specific features in the tumor microenvironment that are usually utilized for the design of bio-responsive drug delivery systems. The numbers in the scheme represent different drug release processes, 1: Degradation, 2: Detachment, 3: Uncap, 4: Charge reversal, 5: Dissociation, 6: Separation.

The tumor microenvironment mainly consists of extracellular matrix, fibroblasts, tumor blood vessels, lymphatic ducts, and immune cells, which reciprocally interact with tumor cells to promote their growth and metastasis (Arneth, 2019; Anderson and Simon, 2020). Due to persistent genetic mutations and particularly rapid proliferation, the tumor microenvironment is characterized by unique pathophysiological indicators compared to normal tissue, such as acidosis, overexpression of special enzymes, hypoxia, and high levels of reactive oxygen species (ROS), GSH, and ATP, all of which can be used as endogenous triggers for the design of responsive DDSs (Vaupel et al., 2019; Thomas et al., 2020; Park et al., 2021). In the current review, these hallmarks of the tumor microenvironment are first summarized in detail, before classifying responsive DDSs into several categories according to these pathological features (Scheme 1). Finally, the potential challenges and future development directions of responsive DDSs are discussed. We believe that this overview will guide the rapid development of this research field.

## 2 Main Pathophysiological Features of the Tumor Microenvironment

Acidosis is the most typical pathophysiological feature of the tumor microenvironment (Ji et al., 2019). The formation of this acidic state is usually attributed to the specific metabolic manner of tumor cells, known as the “Warburg effect,” in which tumor cells produce abundant lactate via the anaerobic glycolytic pathway even in the presence of sufficient oxygen (Hanahan, 2022). Increasing evidence has indicated the acidosis is associated with angiogenesis, tumor metastasis, drug resistance, and immune escape (Damgaci et al., 2018). The hypoxia present in some regions of tumor tissue is another important pathophysiological feature. The speed of angiogenesis cannot fully match the rapid proliferation of tumor cells, which leads to the formation of hypoxic regions in solid tumors (Fukumura and Jain, 2007; Petrova et al., 2018). Besides, as a feedback loop, cancer cells also secrete many proangiogenic factors to promote tumor angiogenesis, which results in disordered growth of the vasculature and further aggravates tumor hypoxia (Schito, 2019). Under hypoxic conditions, tumor cells undergo adaptive gene mutation, such as the acquisition of multidrug resistance, metastasis, suppressed immune response, and angiogenesis, all of which further accelerate tumor progression (Chan and Giaccia, 2007; Rankin and Giaccia, 2016). Growing in such a hostile environment (acidosis, hypoxia), tumor and stromal cells may also overexpress some specific enzymes, including MMP-2, MMP-9, FAP-*α*, legumain, uPA, and some elastases (Wang et al., 1999; Zhang et al., 2019a; Lian and Ji, 2020). Additionally, specific metabolic pathways and regulatory routes induce the excessive production of substances such as ROS, GSH, and ATP (Li et al., 2020a; Deng and Walther, 2020; Tao and Yin, 2020; Cao et al., 2021). Taken together, these unique features of the tumor microenvironment can be reasonably used for the design of stimulus-responsive DDSs. Table 1 summarizes the biological stimuli in the tumor microenvironment and their levels compared to those in normal tissue.

**TABLE 1:** Summary of typical biological stimuli in the tumor microenvironment.

Biological Stimuli	Level in TME	Level in Normal Tissue	Ref
pH	∼6.8	∼7.4	Shin et al. (2021)
ROS	∼100 × 10^−6^M	∼20 × 10^−9^M	Ye et al. (2019)
Enzyme	Different levels according to tumor type	Normal expression	Lian and Ji, (2020)
			Wang et al. (1999)
			Cao et al. (2021)
ATP	100–500 μM	10–100 nM	Sameiyan et al. (2021)
GSH	8–80 μM	2–20 μM	Huo et al. (2014)
Hypoxia	0–2.5 mm High	30–40 mm High	Kumari et al. (2020)

## 3 Internal Stimulus-Responsive DDS

The specific pathophysiological features or differentially expressed substances in the tumor microenvironment provide a possibility for the design of stimulus-responsive DDSs to realize controlled drug release in local tumor tissue (Mura et al., 2013). By doing this, the pharmacokinetic properties, tumor accumulation efficacy, and toxic effects of existing drugs can be reasonably optimized. According to the difference in internal stimulus signals, the following sections summarize the internal stimulus-responsive DDS from six aspects: pH-responsive DDS, ROS-responsive DDS, enzyme-responsive DDS, ATP-responsive DDS, GSH-responsive DDS, and hypoxia-responsive DDS.

### 3.1 pH-Responsive DDS

The lower pH in the tumor microenvironment is probably the most widely used stimulus for the design of responsive DDSs. The design of pH-sensitivity can be realized by the degradation of acid-cleavable bonds or the protonation of ionizable groups (Shin et al., 2021). Thus, we will review pH-responsive DDSs from the following two aspects: pH-responsive DDSs with acid-cleavable bonds, and protonation-based pH-responsive DDSs. The most commonly used acid-sensitive moieties with different responding pH ranges are summarized in Table 2.

**TABLE 2:** Acid-labile moieties and examples. Adapted with permission from (Shin et al., 2021).

Moieties	Nanoparticles	Animal Models	pH Range	Ref
Hydrazone	Hollow silica nanoparticles	HepG2	∼5	Dai et al. (2016)
Acetals	PLLA-based microsphere	4T1	5.0–5.4	Li et al. (2018)
Ketals	Ketal glycoside prodrug nanoparticles	A549	5.0–5.4	Yu et al. (2020)
Ester	Bromelain nanoparticles	H22	∼6	Wang et al. (2020a)
PBAE	Lipid polymer hybrid nanoparticles	4T1	∼6.8	Zhang et al. (2021)
PDMAEMA	PDMS-b-PDMAEMA copolymer nanoparticles	HeLa (*in vitro*)	∼5.5	Car et al. (2014)
Histidine	Star-shaped PLGA nanoparticles	MCF7 (*in vitro*)	∼6.5	Li et al. (2020b)
PEOz	Liposome-platelet membrane hybrid nanoparticles	CT26/4T1	∼6.5	Car et al. (2014)

#### 3.1.1 pH-Responsive DDS With Acid-Cleavable Bonds

Various acid-sensitive bonds have been extensively investigated and used for the design of pH-responsive DDSs for site-specific delivery of different anti-tumor therapeutics. Compared to free drugs, nanomedicine possesses properties such as prolonged circulation time, tumor targeting accumulation, and reduced side effects. Thus, nanoparticles conjugated with chemotherapeutics are a powerful method to optimize the pharmacokinetic properties of free drugs. How to realize controlled drug release in a specific environment, such as the tumor microenvironment and lysosomes, is a popular research area. Hydrazone linkage is one of the most common acid-cleavable bonds and has been widely used in various biological and clinical applications, such as drug conjugation and drug delivery (Sonawane et al., 2017). Bae et al. designed a supramolecular assembly in which doxorubicin was chemically conjugated onto the hydrophobic group *via* hydrazine. They demonstrated that this self-assembling polymeric micelle could release doxorubicin in a pH-dependent manner *in vitro* and function in a highly controlled manner within cells (Bae et al., 2003). Geest’s group developed a pH-sensitive hydrazone-linked Doxorubicin Nanogel (Nanogel^dox^) using polymeric-activated ester as a scaffold (Van Driessche et al., 2018). *In vitro* results revealed that Nanogel^dox^ showed a pH-dependent drug release profile, with enhanced tumor accumulation and tumor growth reduction in a zebrafish embryo tumor model, without obvious systemic toxicity. Zhang’s group constructed a pH-sensitive polymeric complex micelle, which was simultaneously chemically conjugated with DTX via a hydrazine bond and encapsulated with DTX (P123-d–hyd–DTX). The results indicated that P123-d–hyd–DTX showed highly pH-responsive release properties and enhanced anti-tumor activity in an *in vitro* B16F10 cell assay, as well as in an *in vivo* xenograft B16F10 melanoma model, with no obvious adverse effects. Furthermore, P123-d–hyd–DTX exhibited greater tumor cell killing efficacy than commercial drugs (Su et al., 2016). Taken together, these studies revealed the superiority of hydrazone-based nanoparticle-drug conjugations in antitumor applications due to their controlled drug release, enhanced tumor accumulation, antitumor efficacy, and decreased side effects. In addition to the conjugation with chemotherapeutics, hydrazine can also be used for constructing nanoparticles to realize controlled drug release. Cai’s group reported a drug-loading hollow silica nanoparticle (HMSN–PA–HA) whose surface was coated by a hyaluronic acid layer via hydrazine as bridge to block rapid drug release. HMSN–PA–HA could effectively accumulate at tumor sites by HA and CD44 interactions and release loaded drugs when hydrazine linkers between HA agents and HMSNs were broken, triggered by the low pH in the tumor microenvironment (Dai et al., 2016). He’ group constructed a pH sensitive liposomal drug delivery system (Cl-Lip) using PEG5K-Hydrazone-PE for pH responsiveness and DSPE-PEG2K-R8 for cellular endocytosis. Cl-Lip showed a rapid drug release *in vitro* in an acid environment and pH-dependent cellular uptake and cytotoxicity (Zhang et al., 2015). Moreover, similar to hydrazone, imine and oxime linkages with C=N bonds can also be used to construct pH-responsive DDSs. Yang’ group synthesized an amphiphilic polymer (PEG-b-C18) conjugated by a benzoic-imine linker to form an acid-sensitive micelle for drug delivery. Their results verified that the micelle is stable at physiological pH and hydrolyzes under acidic conditions (Ding et al., 2009). Tzakos’ group constructed a peptide-drug conjugate (PDC) using oxime bond as an acid-labile programmable linker. They used gemcitabine as the anticancer unit and gonadotropin releasing hormone (GnRH) as the cancer-targeting unit. The acid-dependent gemcitabine release was monitored *in vitro* in cell culture and in human plasma using LC-MS/MS (Vrettos et al., 2021) Acetals/ketals are also frequently used as acid-sensitive bonds to synthesize prodrugs or pH-sensitive nanoparticles due to their specificity for the acidic environment. Li et al. prepared acetal-based pH-sensitive polymeric microspheres loaded with the anticancer drug doxorubicin hydrochloride. *In vitro* results demonstrated the microspheres showed pH-dependent drug release. Moreover, the results of *in vivo* anticancer experiments indicated that this microsphere showed better antitumor efficiency and prolonged life-span than free doxorubicin, with no obvious cardiotoxicity (Li et al., 2018). Zhong’s group developed 2-[3-[5-amino-1-carboxypentyl]-ureido] pentanedioic acid (Acupa)-decorated pH-responsive chimeric polymersomes (Acupa-CPs) to targeted delivery protein drugs to PSMA-positive LNCaP cells. Meanwhile, an acid-responsive acetal bonds in the vesicular membrane enabled the formed nanocarrier to rapidly release the loaded proteins at mildly acidic pH (Li et al., 2015) Both Zhong’s and Sun’s groups developed acetal-linked paclitaxel prodrug nanoparticles that possessed pH-dependent drug-release properties and enhanced *in vitro* tumor cell killing efficacy (Gu et al., 2013; Zhai et al., 2017). Nishiyama’s group prepared the polymer gemcitabine conjugate through cyclic acetal linkage (P-GEM). P-GEM released GEM in response to acidic environments such as the endosome/lysosome and realized a higher tumor growth suppression effect compared to free GEM, without adverse side effects (Takemoto et al., 2020). Similar to acetals, ketal has also been widely used to construct pH-sensitive nanostructures. Guo’s group developed various ketal-based acid-sensitive prodrugs or nanomedicines, including an amphiphilic etoposide-ketal-glycoside prodrug, which could be fabricated into excipient-free nanoparticles and be activated by the dual action of glycosidase and acid in the lysosome of tumor cells. The authors demonstrated its efficacy in both *in vitro* cell experiments and an *in vivo* A549 xenograft tumor model (Yu et al., 2020). They also chemically conjugated the anticancer agent paclitaxel with different lengths of poly (ethylene glycol) (PEG) via an acyclic-ketal linkage to form an amphipathic structure that self-assembled into acid-activated nanoparticles (PKPs). The *in vitro* and *in vivo* results demonstrated that PKP nanoparticles showed acid-dependent drug release, improved pharmacokinetics, and superior antitumor efficacy (Mu et al., 2020). They also conjugated the anticancer nucleoside drug gemcitabine to the polyketal backbone via pH-sensitive ketal linkage and prepared pH-sensitive prodrug nanoparticles (Zhong et al., 2020). Orthoester has been widely applied for constructing pH-sensitive nanomedicines for cancer therapy due to its good biocompatibility. Tang’s group developed a pH-triggered hyaluronic acid nanogel system (HA-NGs) using orthoester as a linkage. HA-NGs exhibited pH-dependent degradation and drug release *in vitro*, enhanced tumor killing efficacy, and excellent tumor homing capability due to hyaluronic acid and CD44 interaction (Yang et al., 2017a). They also developed acid-degradable carboxymethyl chitosan nanogels, bromelain nanoparticles, and chitosan nanogels via an orthoester linkage, all of which were shown to have pH-triggered drug-release properties and improved tumor cell killing (Zha et al., 2017; Li et al., 2019a; Wang et al., 2020a; Wang et al., 2021). *ß*-thiopropionate, containing ester bonds, is also used for the preparation of acid-labile polymers. Chen’s group synthesized a biodegradable PAMAM-OH derivative (PAMSPF) containing an acid-labile b-thiopropionate bond for gene delivery. Approximately 95.8% of the loaded DNA in PAMSPF was rapidly released after incubation under acidic conditions. PAMSPF exhibited high transfection efficiency in KB and HepG2 cell lines (Chen et al., 2016). Acidosis is a universal feature of the tumor microenvironment and is suitable for the design of nanomedicines with controlled drug release features against almost all kinds of cancer.

#### 3.1.2 Protonation-Based pH-Responsive DDS

**FIGURE 1:**
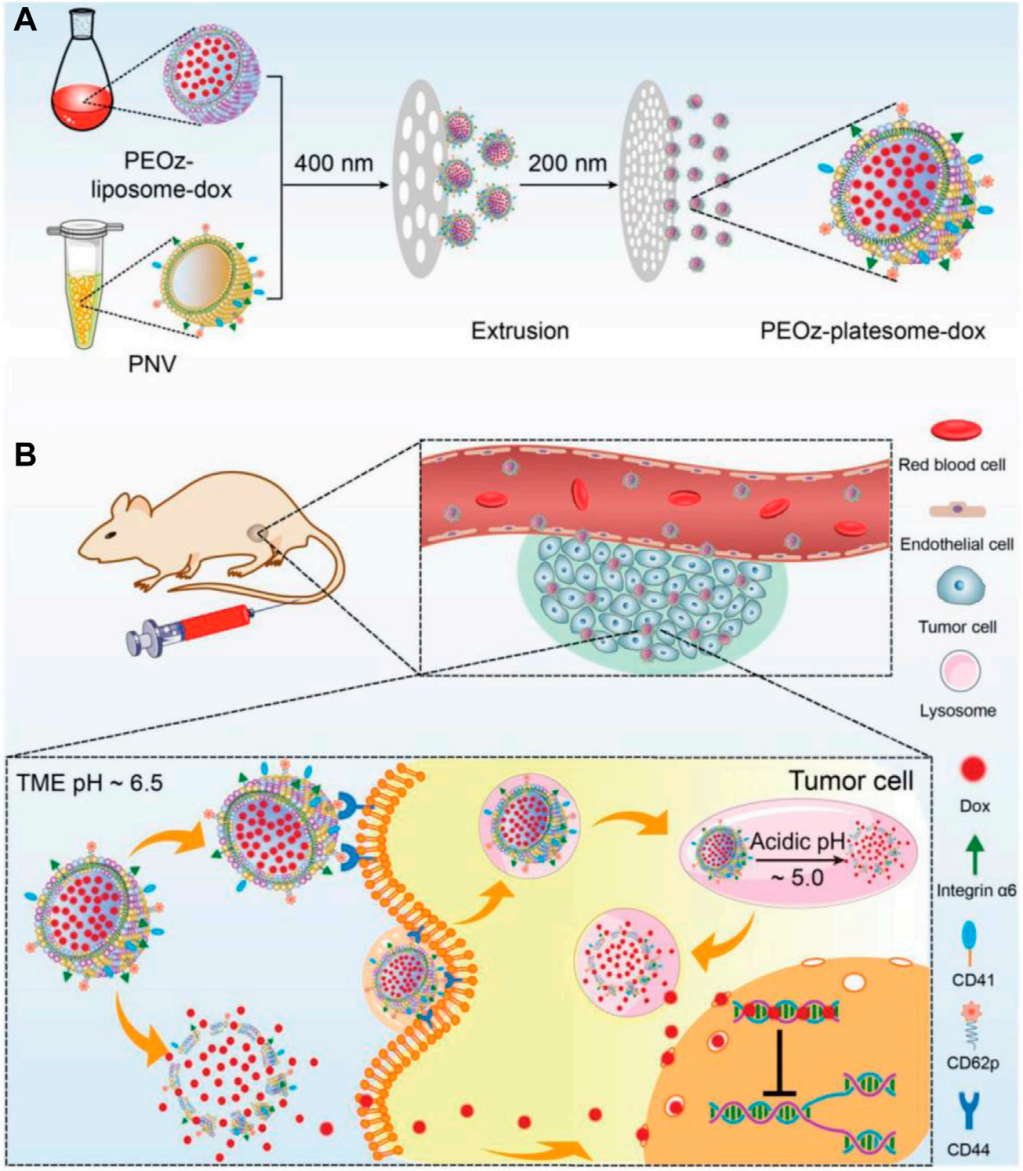
Schematic illustration of the preparation of PEOz-platesome-dox **(A)** PEOz-platesome-dox was generated by coextrusion of PEOz-liposome-dox and PNV (platelet membrane nanovesicles). **(B)** After intravenous injection, PEOz-platesome-dox is expected to target the tumor through molecular interactions between platelet membrane and tumor cell substituents, such as platelet CD62p and its cognate receptor, tumoral CD44. The incorporation of pH-sensitive lipids into the platesome would allow its cargo (dox) to be rapidly released at the tumor site in response to the acidic pH of the tumor microenvironment and/or lysosomal compartments. The released dox kills tumor cells by inhibiting the cellular DNA replication. Adapted with permission from (Liu et al., 2019).

In addition to pH-sensitive chemical bonds, materials with de/protonation properties under different pH conditions can also be used for the design of pH-responsive nanocarriers, e.g., poly (*β*-amino ester) (PBAE), poly (2-(dimethylamino) ethyl methacrylate) (PDMAEMA), poly (histidine) (poly (His)), and poly (2-ethyl-2-oxazoline) (PEOz). Wang’s group developed a pH-responsive lipid polymer hybrid nanoparticle (FA/PBAE/DTX-NPs) consisting of two portions, a docetaxel-loaded polymer inner core assembled from poly (β-amino ester), and a lipid outer shell of DSPE-PEG2000, FA-DSPE-PEG2000, and lecithin. The *in vitro* results indicated that FA/PBAE/DTX-NPs can efficiently target to 4T1 tumor cells and release drugs in a pH-dependent manner on demand. The *in vivo* results also showed that FA/PBAE/DTX-NPs possessed tumor targeting ability, significant antitumor effects, and minimal systemic toxicity in tumor-bearing mice (Zhang et al., 2021). Poly (*β*-amino ester) can also be used as a hydrophobic chain to construct amphipathic polymer, which can self-assemble into micelles and disassemble under acidic conditions. Li et al. synthesized a PEG-Poly (-Amino Ester) copolymer to develop a pH-responsive drug-loaded micelle (Thz/PPM), which was shown to be an effective pH-responsive DDS (Li et al., 2020b). Lin’s group synthesized a triblock copolymer MPEG-b-PBAE-b-PLA to construct a pH-responsive drug-loaded micelle (Yang et al., 2018). PDMAEMA was also used as pH-responsive block to realize controlled drug release. Meier’s group prepared a PDMS-b-PDMAEMA block copolymer via atom transfer radical polymerization, which could self-assemble into micelles in solution and encapsulate chemotherapeutic doxorubicin, with a pH-dependent drug release feature (Car et al., 2014). Tang’s group covered PDMAEMA polymer on the surface of drug-loaded hollow mesoporous silica nanoparticles to control drug release. Histidine has been reported as a pH-responsive element due to its pKa of ∼6.5 (Yu et al., 2013). Roy’s group developed a histidine modified star-shaped PLGA (sPLGA-His) to co-deliver the chemotherapeutics docetaxel and disulfiram. sPLGA-His was demonstrated to have pH-dependent drug release, lysosome escape, deep tumor penetration, and enhanced tumor cell killing (Swetha et al., 2021). Gu’s group synthesized an amphiphilic triblock copolymer methoxy-poly (ethylene glycol)-poly (l-histidine)-poly (l-lactide) (mPEG-PH-PLLA) to form pH-sensitive nanoparticles (Liu et al., 2012). The changes in the diameter and surface charge revealed that such copolymer nanoparticles have a pH-responsive feature. As a potential replacement for PEG, PEOz (poly (2-ethyl-2-oxazoline)) has its own advantages, including good stability and biocompatibility, higher reactive group substitution rate and pH-responsiveness. Liu et al. developed a platelet membrane–lipid hybrid DDS (platesome) by co-extrusion of pH-responsive Dox-loaded liposomes and platelet membrane nanovesicles, in which pH-responsiveness was realized by insertion of pH-sensitive lipid DSPE-PEOz. The introduction of platelet membrane endowed the nanoparticles with tumor targeting capability (Figure 1). Their results demonstrated that the platesome could release drugs in a pH-dependent manner and efficiently target and kill tumor cells (Liu et al., 2019). Wang et al. prepared a PEOz-b-PLA micelle and investigated its possible mechanisms of pH-sensitivity and cellular internalization in detail. They attributed the pH-responsiveness to the ionization of the tertiary amide groups along the PEOz chain at pH lower than its pKa (Wang et al., 2017). Li’s group used the polymer material poly (2-ethyl-2-oxazoline)-poly (l-lactide) (PEOz-PLA) to prepare self-assembled micelles, which showed pH-dependent drug release *in vitro* (Su et al., 2019). Xu et al. synthesized a novel material poly (2-ethyl-2-oxazoline)-cholesterol hemisuccinate (PEtOz-CHEMS) which will change from hydrophilic to hydrophobic under acidic conditions and can be used for the construction of pH-sensitive liposomes. They prepared the dox-loaded pH-responsive liposomes (PEtOz-L) and verified its pH-induced increase in particle size and drug release (Xu et al., 2015). For the protonation-based pH-responsive DDS, the responsiveness not only realizes spatially and/or temporally controlled drug release but also has the possibility of improving lysosome escape for enhanced antitumor activity. In addition, polyelectrolyte-based nanocarriers also play an important role in development of pH-responsive DDS. Suo’ group developed the Doxorubicin/cisplatin co-loaded hyaluronic acid/chitosan-based nanoparticles that exhibited pH-dependent drug release *in vitro* and improved synergistic anti-cancer activity (Wang et al., 2019a). Liu’s group prepared chitosan-based micelles (DOX/CS-PEG) for the intracellular delivery of anti-cancer drug doxorubicin to realize pH-responsive drug release in a lysosome environment. DOX/CS-PEG achieved a cumulative release ratio of 72.76% at pH 5.0 and only 2.32% at pH 7.4 *in vitro* and showed significant tumor cell killing efficacy (Zhao et al., 2016).

Acidity is the most basic feature for most tumors. In the tumor microenvironment, there are obvious pH gradients (pH = ∼6.8 in the TME and pH = 4–5 in lysosomes) in favor of the design of DDSs for delivering different drugs with different mechanisms of action. However, the acidity of tumors varies widely in different tumors or different stages of the same tumor. Thus, the designed pH-responsive DDS is difficult to have universal applicability. The evaluation of pH levels in different tumors or different stage of the same tumor to establish a standard database is a promising approach to overcome above-mentioned problems.

### 3.2 ROS-Responsive DDS

Asides from the acidic conditions, the tumor microenvironment also overproduces ROS, which can also be used as a stimulus to design a responsive DDS. Massive efforts have been made in the development of ROS-responsive drug-release systems, most of which are based on thioether-based materials, diselenide linkages, thioketal-based materials, boronic ester-based, and polyoxalate-based materials (Ye et al., 2019; Cao et al., 2021). The typical examples of ROS-responsive DDS are summarized in Table 3.

**TABLE 3:** ROS-responsive DDS and examples.

Linkages	Drug Formulations	Release Mechanisms	Evaluation Method	Cell Lines or Animal Models	References
Thioether	Copolymer micelles	hydrophobic to hydrophilic transition	*In vitro* drug release	MDA-MB-231	Wang et al. (2019b)
Thioether	Liposome nanoparticles	hydrophobic to hydrophilic transition	DLS, TEM, UV−vis, Spectrometry, *In vitro* drug release	A549, MCF7, 4T1tumor mice model	Du et al. (2019)
Diselenide	Polymer aggregates	cleavage	DLS, TEM, GPC, XPS and FTIR	MDA-MB 231	Sun et al. (2017)
Diselenide	Diselenide–pemetrexed assemblies	cleavage	DLS, TEM, ESI-MS, NMR, XPS, FTIR	MDA-MB-231, MDA-MB 231 tumor mice model	Li et al. (2020c)
Diselenide	Mesoporous silica nanoparticles	cleavage	TEM, XPS, *In vitro* degration, *In vitro* drug release	HeLa, RAW264.7, MCF7, HeLa tumor mice model	Shao et al. (2018)
Diselenide	Polymeric micelles	cleavage	*In vitro* drug release	PC3, PC3 tumor-bearing mice	Deepagan et al. (2016)
Thioketal	Polyphosphoester-doxorubicin conjugate	cleavage	*In vitro* degration, *In vitro* drug release	MDA-MB-231, MDA-MB 231 tumor mice model	Pei et al. (2019)
Thioketal	Prodrug nanoparticles	cleavage	Digital photos, MALDI-TOF MS, DLS and TEM	HepG2, HepG2 tumor mice model	Pan et al. (2020)
Thioketal	Prodrug nanoparticles	cleavage	GPC, HPLC, *In vitro* drug release	PC3, DU145, 22RV1, and LNCaP, LNCaP tumor mice model	Xu et al. (2017)
Polyoxalate	Prodrug nanoparticles	hydrolysis	cryo-TEM, NMR, DLS	LNCaP, PC3	Höcherl et al. (2017)
Boronic Ester	Lipid-polymer hybrid nanoparticles	hydrolysis	NMR, Agarose gel electrophoresis	SW620, SW480, SW620 tumor mice model	Li et al. (2017a)

Thioether-based materials achieve ROS-responsiveness through hydrophilic-hydrophobic transformation in structure. Since Hubbell’s group first copolymerized oxidation-convertible poly (propylene sulfide) (PPS) with polyethylene glycol (PEG) to form nanovesicles for drug delivery, various ROS-responsive DDSs have been developed (Napoli et al., 2004). Hasegawa’s group synthesized three micelles containing different thioether groups (3-methylthiopropylamide (TPAM), thiomorpholine amide (TMAM), and 4-(methylthio) benzylamide (TPhAM)) within the core to form TP, TM, and TPh micelles, respectively. They compared the stability of the three micelles in human liver cancer (HepG2) cells and human umbilical vein endothelial cells (HUVECs) and demonstrated that the TP micelles showed the highest oxidation sensitivity, while the TPh micelles presented the lowest reactivity toward H_2_O_2_. They also investigated the cytotoxicity of these three micelles after dox loading and reached a result that was consistent with the oxidation sensitivity (van der Vlies et al., 2022). He’s group also constructed three ROS-responsive amphiphilic copolymers of mPEG-poly (ester-thioether), mPEG-poly (thioketalester), and mPEG-poly (thioketal-ester-thioether), and three fabricated polymeric nanoparticles using thioketal and thioether, two moieties with ROS sensitivity. They demonstrated the ROS-responsive behavior of the three nanoparticles by NMR, DLS, SEM and *in vitro* drug release profiles, and demonstrated that all of them showed enhanced cellular uptake and anticancer efficacy (Xu et al., 2019). Wang’s group synthesized an amphiphilic copolymer consisting of polyethylene glycol and polyphosphoester with thioether groups in the side chain (mPEG-b-PMSPEP), self-assembling into nanoparticles with sensitivity for H_2_O_2_. They co-encapsulated the photosensitizer chlorin e6 (Ce6) and anticancer drug paclitaxel (PTX) into PMSPEP nanoparticles and realized photo-accelerated release of PTX *in vitro*. The cytotoxicity test also demonstrated the enhanced tumor cell killing efficacy of PMSPEP nanoparticles compared to photodynamic therapy (Wang et al., 2019b). Aside from polymeric nanoparticles, lipid-based nanoparticles are one of the most applied nanocarriers. Du et al. developed a series of phosphatidylcholine-containing thioether moieties (S-PCs) with different chain lengths. Using these phosphatidylcholines, they prepared S-PC-based stealth liposomes and investigated their ROS responsiveness *in vitro* and *in vivo*, showing enhanced tumor cell killing efficacy in comparison with nonresponsive liposome (Du et al., 2019).

**FIGURE 2:**
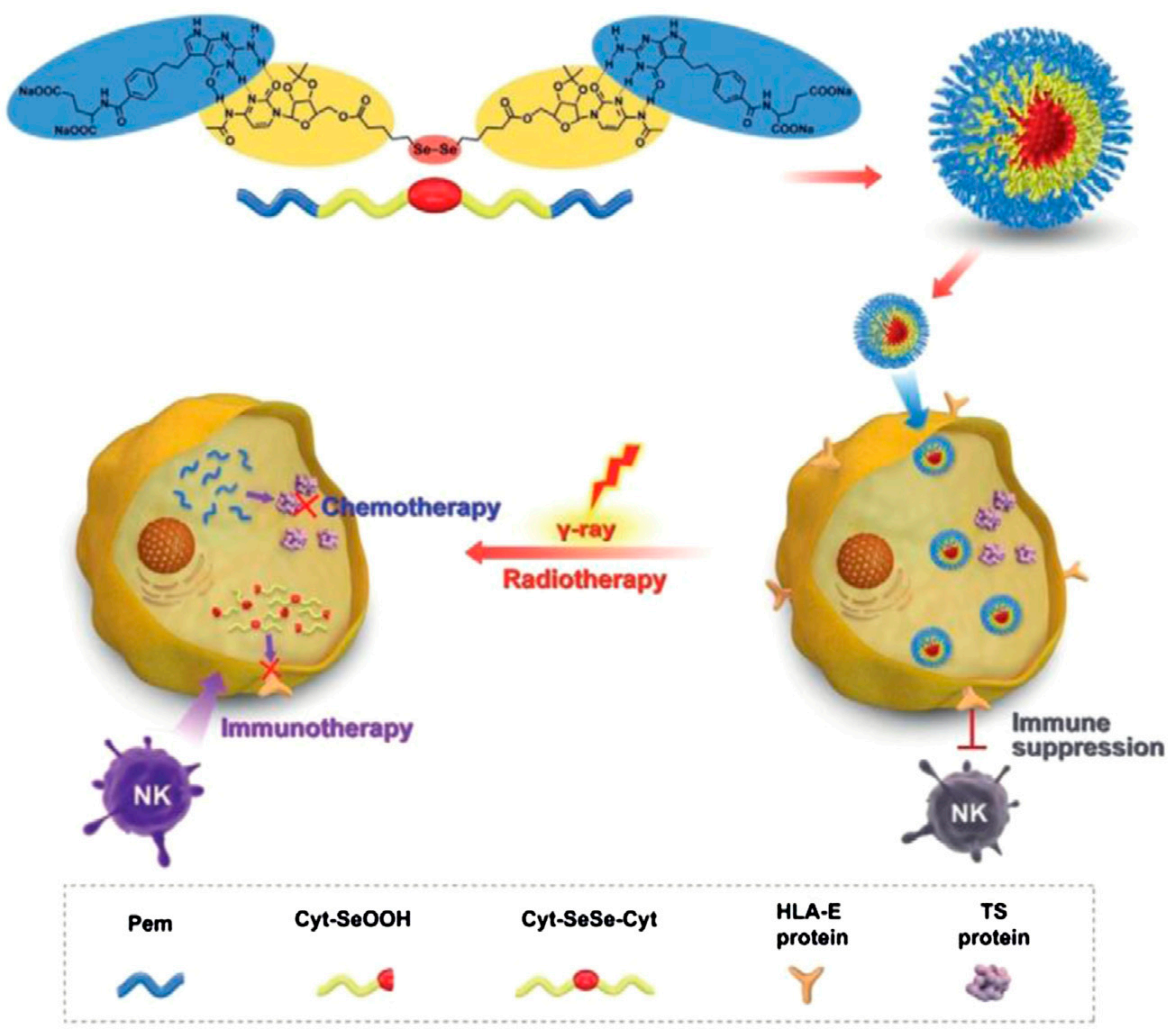
Schematic illustration of diselenide-pemetrexed assemblies for combined cancer immunotherapy with radiotherapy and chemotherapy. Under *γ*-radiation, diselenides are transformed into seleninic acid, pemetrexed is released from the assemblies, and combination therapy including immunotherapy, radiotherapy and chemotherapy is consequently achieved. Adapted with permission from (Li et al., 2020c).

Another important ROS-sensitive motif used for engineering-responsive nanomedicine is the diselenide linkage, which also shows specific biological functions. Xu’s group developed various diselenide-based nanostructures for cancer therapy, one of which was a diselenide/porphyrin-containing hyperbranched polymer that self-assembled into a nanostructure (PSe-Por) in solution. When PSe-Por was exposed to visible light, its structure depolymerized; surprisingly, the oxidized products presented cytotoxicity to cancer cells without the help of any cytotoxic molecule (Sun et al., 2017). In another work, they demonstrated that diselenide in diselenide–pemetrexed assemblies could be oxidized to seleninic acid, which showed the capability to suppress the expression of human leukocyte antigen E (HLA-E) in cancer cells for immune therapy (Figure 2). Besides, the produced seleninic acid also suppressed the expression of vascular endothelial growth factor (VEGF) and matrix metalloproteinase-2 (MMP-2) to achieve anti-angiogenesis therapy (Li et al., 2020c). Lang’s group also showed that diselenide-based polymers without drugs (PSeSeTMC) had high therapeutic efficacy in a controlled manner by regulating the contents of diselenide with no obvious side effects (Wei et al., 2021). Shao and co-workers incorporated diselenide linkage into mesoporous silica nanoparticles for X-ray and ROS responsiveness. They used this nanosystem to deliver chemotherapeutics and protein drugs and realized controlled drug release for cancer therapy (Shao et al., 2018; Shao et al., 2020). Park’s group developed diselenide-crosslinked and dox-loaded micelles (dox-DCMs) using polyethylene glycol (PEG) and polypeptide derivatives as copolymer building components to realize controlled drug release in the tumor site. The *in vivo* anti-tumor results indicated that dox-DCMs could significantly accumulate in tumor tissue and suppress tumor growth (Deepagan et al., 2016).

Thioketal linkage is one of the most important ROS-cleavable motifs used for constructing ROS-responsive nanomedicines or prodrugs. Wang’s group developed a ROS-activatable drug-polymer conjugate in which the chemotherapeutic doxorubicin (dox) was chemically conjugated to the polyphosphoesters (PPEs) via a thioketal bond (PPE-TK-dox). Then, the photosensitizer Ce6 was encapsulated into the self-assembly of PPE-TK-dox in solution to obtain Ce6@PPE-TK-dox. When Ce6@PPE-TK-dox in tumor tissue was illuminated under 660-nm red light, the produced ROS cleaved the thioketal bond and triggered the rapid release of dox to kill tumor cells (Pei et al., 2019). Using a similar strategy, Pan et al. prepared a PEG doxorubicin (PEG-dox) prodrug using a thioketal moiety as the linker, which also self-assembled into nanoparticles in solution. The *in vitro* results indicated that the synthesized nanoparticles showed ROS-responsive properties (Pan et al., 2020). Farokhzad’s group developed a ROS-responsive polyprodrug by crosslinking the anticancer drug mitoxantrone (MTO) *via* a thioketal moiety as the linker, self-assembling with a lipid structure to form polyprodrug NPs (polyMTO). Simultaneously, a tumor targeting peptide iRGD was modified onto the surface of polyMTO. The results revealed that polyMTO NPs showed a ROS-dependent release of intact therapeutic drug molecules and enhanced anti-cancer efficacy (Xu et al., 2017). Thioketal linkage is also widely used for constructing prodrugs due to their high sensitivity and stability (Chu et al., 2020; Hao et al., 2020). Polyoxalate-based nanoparticles were originally designed for the imaging of H_2_O_2_ due to their high selectivity and sensitivity. Only a few of them have been applied for tumor therapy. Höcherl and co-workers developed a novel self-immolative H_2_O_2_-sensitive polyprodrug material (PDEB NPs) through a one-pot synthesis method. PDEB NPs showed H_2_O_2_-dependent structure degradation and released chemotherapeutic drugs for enhanced tumor cell killing efficacy (Höcherl et al., 2017). In addition to the above-mentioned examples, other ROS-sensitive motifs, such as boronic ester, have also been used as bio-responsive materials for drug delivery in cancer therapy. Indeed, Ping’s group developed a ROS-responsive boronic vehicle with a lipid layer on the surface for siRNA delivery. This system not only protected siRNA in circulation from degradation, but also achieved the efficient systemic transportation of siRNA to the tumor site for effective knockdown of the targeted gene *in vivo* (Li et al., 2017a).

Elevated ROS level is not only an important feature in the tumor microenvironment but also a toxic substance that can induce the apoptosis of tumor cells. Therefore, the strategy inducing the production of ROS to kill tumor cells, e.g., photodynamic therapy, is suitable for the combination with ROS-responsive DDSs. The main limitation of this kind bio-responsive nanoparticle is the complicated manufacturing process which hinders its large-scale production and clinical translation. It is very important to develop response motifs with simple structure and easy preparation. In addition, the heterogeneity of ROS concentration is also an important challenge.

### 3.3 Enzyme-Responsive DDS

Enzymes play important roles in almost every step of cancer progression, including angiogenesis, cell proliferation, and metastasis (Muz et al., 2015; Conlon and Murray, 2019). In the specific tumor microenvironment, various enzymes are highly expressed in tumor tissue but have relatively low expression in normal tissues, which offers the possibility for the design of enzyme-responsive DDSs (Shahriari et al., 2019; Li et al., 2020d). The typical enzyme-responsive DDSs and examples are summarized in Table 4.

**TABLE 4:** Enzyme-responsive DDS and examples.

Enzyme	Sensitive Sequences or Moieties	Drug Formulations	Cell Lines and Animal Models	References
MMP2	DSK(C18)DSGPLGIAGQDSK(C18)	Lipid-polymer hybrid nanoparticles	MCF7, A549, HUVEC, MCF7 tumor animal model, A549 tumor mice model	Li et al. (2017b)
MMP2	GPLGIAGQ	Liposome nanoparticles	B16F10, 4T1, NIH/3T3, HeLa, A549, MCF-7, HepG2, or RAW264.7, 4T1 tumor mice model	Liu et al. (2020)
MMP2	PVGLIGG	Mesoporous silica nanoparticles	HepG2, Raw264.7, HepG2 tumor mice model	Liu et al. (2015)
MMP2	CGPLGVRGGGGYEQDPWGVKWWYGGGS-KLAKLAKKLAKLAK	Hyaluronic acid modified gold nanorod	B16F10, M1 and M2 macrophages, B16F10 tumor mice model	Tian et al. (2021)
MMP2	NA	Gelatin nanoparticles	4T1, 4T1tumor mice model	Li et al. (2021)
MMP2	PLG-LAG	Core-shell Micelleplex	MDA-MB-231, HT-1080, HEK293, MDA-MB-231 tumor mice model	Wang et al. (2014)
MMP9	GPLGLPG	Liposome nanoparticles	LS180, HeLa, LS180 tumor mice model	Han et al. (2021)
MMP9	RSWMGLP	Mesoporous silica nanoparticles	A549, H1299, 129S/Sv-Kras^tm3Tyj^/J (K-ras^LA2^) mutant mice	van Rijt et al. (2015)
MMP9	CCVVGRKKRRQRRRPQGGPLGVEKEKEKEK	Gold nanorods	HepG2, HepG2 tumor mice model	Wu et al. (2019)
Cathepsins-B	GIVRAK	Mesoporous Silica Nanoparticles	HeLa, MEFs	de la Torre et al. (2014)
Cathepsins-B	GFLG	Liposome nanoparticles	Hep G2, Hep G2 tumor Zebrafish model	Lee et al. (2020)
Cathepsins-B	GFLG	Dendrimer nanoparticles	CT26, CT26 tumor mouse model	Lee et al. (2015)
Cathepsins-B	GFLG	Dendrimer nanoparticles	4T1, 4T1tumor mouse model	Dai et al. (2018)
Cathepsins-L	Lys (Ac)-Puro	Prodrugs	HCT116 tumor mouse model	Ueki et al. (2013)
PSA	Nglutaryl-(4-hydroxyprolyl) AlaSer-cyclohexaglycylGlnSerLeu-CO2H	Prodrugs	LNCaP, DuPRO-1, PC3, Hct116, MDAMB435S, T24, MRC-5, LNCaP, CWR22 and DuPro-1 tumor animal models	DeFeo-Jones et al. (2000)
Urokinase	LGGSGRSANAILEC	Gold nanorods	4T1tumor mouse model	Niidome et al. (2010)
Legumain	ANN	Peptide nanoparticles	SVEC4-10, RAW264.7, 4T1tumor mouse model	Zhang et al. (2019a)

**FIGURE 3:**
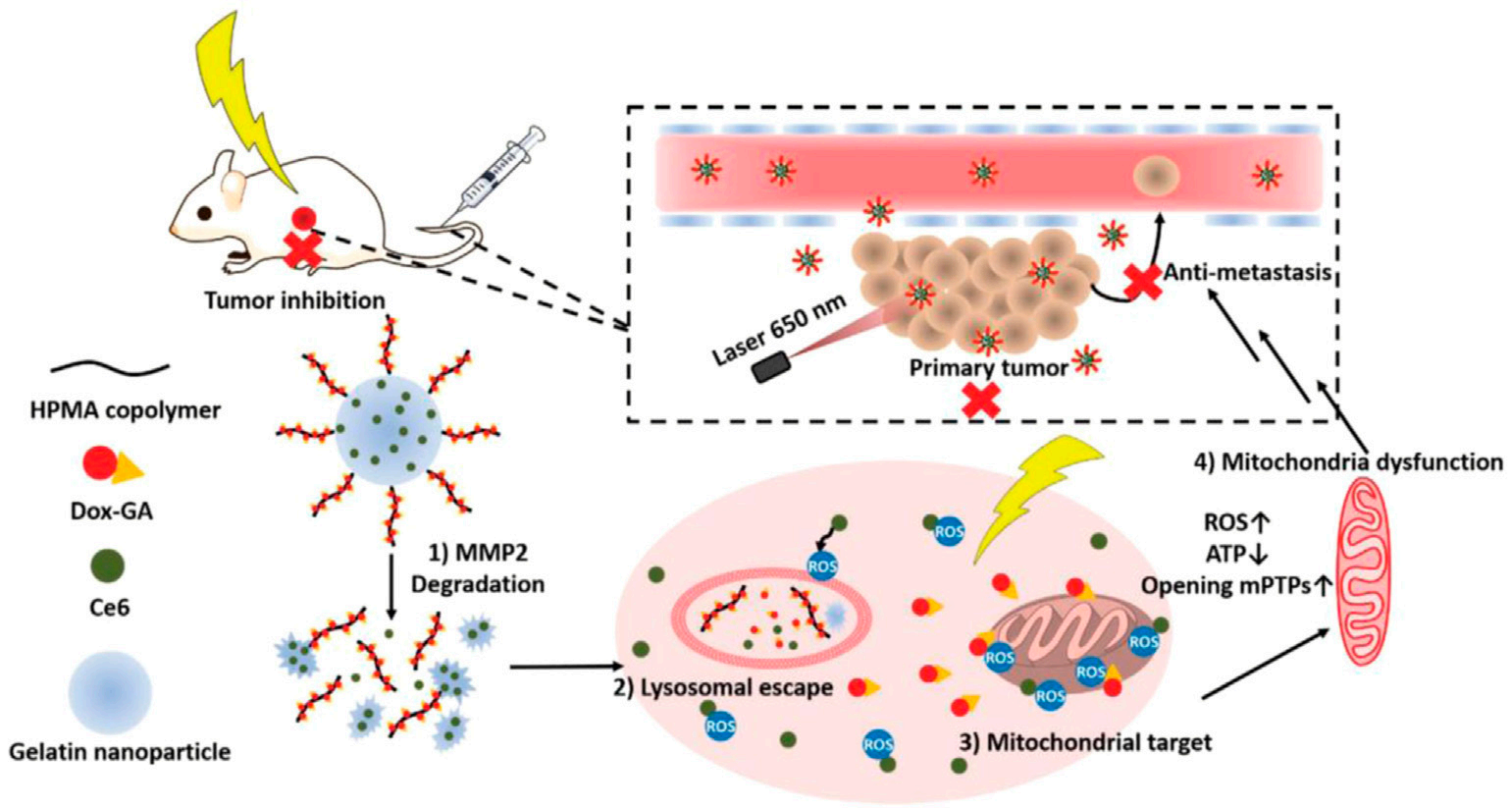
Schematic illustration of the stimuli-responsive nanoparticles combining photodynamic therapy and mitochondrial disruption to suppress tumor metastasis. When the nanoparticles arrived at tumor site, they collapsed in response to highly expressed matrix metallopeptidase 2 (MMP2) and released the Ce6 and polymer–Dox–GA conjugates. Then, Dox–GA was detached from the polymer in tumor lysosomes and escaped by Ce6-mediated photodynamic therapy under irradiation. Finally, Dox–GA subsequently targeted mitochondria to reduce the O2 level to maximize the efficacy of PDT. Adapted with permission from (Li et al., 2021)

Matrix metalloproteinases (MMPs), mainly including MMP-2 and MMP-9, which are overexpressed in the tumor microenvironment, have long been closely implicated in tumor invasion and metastasis and can be applied as stimuli for the design of bio-responsive materials (Yao et al., 2018). Li et al. inserted an amphiphilic peptide with an MMP-2-cleavable site into the structure of the lipid bilayer to package the surface of dox-loaded nanoparticles. When the lipid-polymer hybrid nanoparticles enter the tumor tissue by EPR effects, the lipid layer is degraded by the overexpressed MMP-2 and the platelet antibody and dox-loaded inner core are released to mediate effects at different regions of the tumor microenvironment (Li et al., 2017b). Besides, peptides with an MMP-2-cleavable sequence are frequently used to link functional elements on the surface of nanoparticles to realize controlled release or exposure. Liu et al. prepared an MMP-2-sensitive PS-modified nanoparticle in which PS could be exposed and mediate the phagocytosis of tumor-associated macrophages (TAMs) when PEG coating was specifically cleaved by the overexpressed MMP-2 in the tumor site (Liu et al., 2020). Moreover, Cai’s group kinked phenylboronic acid conjugated human serum albumin (PBA-HSA) onto the surface of mesoporous silica nanoparticles (MSNs) via a MMP-2 peptide to realize controlled drug release (Liu et al., 2015). Furthermore, Ai’s group developed a hyaluronic acid-modified gold nanorod (HA-AuNR) surface-linked with an M2-type TAMs (M2-TAMs) depletion peptide via an MMP-2-sensitive peptide, termed HA-AuNR/M-M2pep. This combined PTT and M2-TAMs depletion strategy effectively inhibited tumor growth (Tian et al., 2021). Inspired by the natural substrates for enzymes found in living organisms, researchers have also developed many nanomedicines with intrinsic enzyme responsiveness using these substrates. Indeed, Huang’s group established an MMP-2-responsive nanoparticle using gelatin as the basic framework (Li et al., 2021). The nanoparticle (GD/Ce6@GNP) consists of a gelatin core encapsulating photosensitizer chlorin e6 (Ce6) and a polymeric shell chemically conjugated to mitochondria-targeted anticancer drugs (doxorubicin-glycyrrhetinic acid conjugates) on the surface. When arriving at tumor tissue through the EPR effect, GD/Ce6@GNP was significantly degraded, and the Ce6 and doxorubicin-glycyrrhetinic acid conjugates were released. Moreover, the results showed that O_2_ consumption induced by mitochondria targeting significantly enhanced the effect of PDT (Figure 3). An MMP-2-cleavable peptide can also be used as the linkage between the hydrophilic group and hydrophobic group of amphiphilic lipids or polymers for constructing nanoparticles. Wang et al. synthesized a block copolymer containing PEG, MMP-2-degradable peptide, cationic cell penetrating peptide polyarginine r9, and poly (ε-caprolactone) (PCL) to prepare a core-shell for siRNA delivery. The decoration of PEG endowed the nanostructure with prolonged circulating time in blood. When the nanoparticle accumulated at the tumor site, the PEG layer was cleaved and the exposed cell penetrating peptide R9 enhanced cellular uptake of siRNA (Wang et al., 2014). MMP-9 is another matrix metalloproteinase overexpressed in tumors that is used for the design of responsive nanomedicines. Surface modification of nanoparticles by hydrophilic moieties using responsive linkers is often used to optimize pharmacokinetic properties, prevent drug leakage in the circulation, and realize surface charge changes to enhance cellular uptake in the tumor area. Zhang’s group constructed an OMPE (glutamate-rich segment) modified cationic liposome (O-NP) using MMP-9-cleavable peptide as linker. The surface change of O-NP changed from positive to negative upon binding MMP-9 in tumor tissue, which results in the enhanced endocytosis of tumor cells (Han et al., 2021). Meiners’s group packaged mesoporous silica nanoparticles (MSNs) using avidin molecules via MMP-9 sensitive linkers and realized spatiotemporally controlled release of loaded cargoes. They demonstrated the effectiveness of their proof-of-concept design in vitro and *in vivo* experiments (van Rijt et al., 2015). Gao’s group modified the gold nanorods via an MMP-9-responsive zwitterionic stealth peptide consisting of a cell penetrating Tat sequence, an MMP-9 cleavable sequence, and a zwitterionic antifouling sequence. This modification can improve the systemic circulating time and significantly enhance cellular uptake post-cleavage by MMP-9 in the tumor microenvironment (Wu et al., 2019). Mallik’s group synthesized a collagen mimetic lipid-peptide hybrid nanovesicle in which the MMP-9-cleavable peptide was incorporated to realize controlled drug release (Kulkarni et al., 2014). Additionally, other MMPs, including MMP 7, MMP12, MMP13, and MMP14, can also be used for the design of controlled drug delivery systems (Zhang et al., 2019b).

Cathepsins are highly associated with cancer development and metastasis and can be used as the stimuli of responsive nanoparticles. Torre et al. synthesized the silica mesoporous nanoparticles, the surface of which was capped by cathepsin B-responsive peptide to realize controlled release of loaded cargoes. They verified their design by encapsulating safranin O or doxorubicin into the nanoparticles (de la Torre et al., 2014). Choi’s group chemically linked the hydrophilic group PEG with the hydrophobic group via a cathepsin B-responsive peptide to self-assemble into liposomes along with DOTAP (1,2-dioleoyl-3-trimethylammonium-propane (chloride salt)), DPPC (dipalmitoylphosphatidylcholine), and cholesterol. The prepared liposomes showed cathepsin B-dependent drug release and inhibited cancer cell proliferation in a zebrafish model (Lee et al., 2020). Besides, dendrimers have been extensively investigated in the biomedical field due to their high biocompatibility, easy modifiability, and drug loading capability. Lee’s group conjugated the chemotherapeutic dox with dendrimer-methoxy poly (ethylene glycol) (MPEG) via a cathepsin B-cleavable peptide to form responsive nanoparticles (Lee et al., 2015). Luo’s group also conjugated gemcitabine with a polyHPMA copolymer *via* a cathepsin B- cleavable peptide to form stimuli-responsive dendritic systems (Dai et al., 2018). Aside from cathepsin B, other cathepsins can also be used for constructing responsive DDSs, including cathepsin E and cathepsin L (Abd-Elgaliel et al., 2013). Hayman’s group developed a prodrug by coupling an acetylated lysine group to puromycin which can be activated by the endogenous protease cathepsin L. *In vivo* anti-tumor experiments indicated that the designed prodrug exhibited efficient tumor growth inhibition in cancer models with high cathepsin L activity (Ueki et al., 2013). Prostate-specific antigen (PSA), a type of serine protease that only exists around the prostate cells, is significantly overexpressed in the tumor microenvironment and can be used for the design of enzyme-responsive prodrugs or nanomedicines. Jones’s group first covalently linked doxorubicin with a peptide that is hydrolyzable by prostate-specific antigen to form an anti-cancer prodrug. This prodrug showed much cytotoxic activity in prostate-specific antigen overexpressing cell lines and tumor models (DeFeo-Jones et al., 2000). Niidome et al. modified gold nanorods using PEG chains by a peptide linker, a substrate for urokinase-type plasminogen activator (uPA). After arriving at tumor tissue with high uPA expression, the nanorods effectively released the PEG chain (Niidome et al., 2010). To overcome the pharmacokinetic deficiencies of a small peptide with tyrosine kinase with immunoglobulin and epidermal growth factor homology-2 (Tie2) inhibiting effects, Zhang et al. designed a dual-responsive amphiphilic peptide that self-assembled into nanoparticles in solution. The formed nanoformulation (P-T4) releases the small peptide at the tumor local site in response to legumain, which is commonly overexpressed in tumor tissue and inhibited tumor growth and metastasis (Zhang et al., 2019a). Additionally, trypsin, phospholipases and peroxidase can be used as triggers of responsive nanomedicines (Lu et al., 2016; Dou et al., 2020).

Tumor-associated enzymes are tumor-specific and highly associated with the type and stage of cancer. Future clinical applications require precise design and treatment. In addition, the enzyme activity assay within tumor tissue is also an important research field. The greatest challenge enzyme-responsive DDS faced is the complicated process for the introduction of enzyme-cleavable peptides. Besides, differences in enzyme expression between animal models and clinical tumor patients also need to be considered.

### 3.4 ATP-Responsive DDS

**FIGURE 4:**
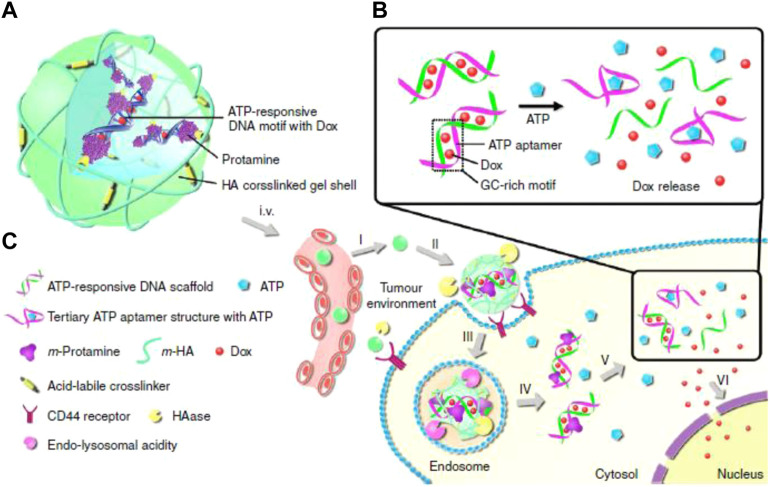
Schematic illustration of the ATP-triggered dox release system **(A)** The main components of Dox/NG: ATP-responsive DNA motif with Dox, protamine and a HA-crosslinked gel shell. **(B)** Mechanism of ATP-triggered release of Dox based on the structural change of duplex-to-aptamer. **(C)** ATP-responsive delivery of Dox by Dox/NG to nuclei for the targeted cancer therapy. I, accumulation of Dox/NG at the tumor site through passive and active targeting; II, specific binding to the overexpressing receptors on the tumor cells and degradation of HA shell by HAase rich in the tumor extracellular matrix; III, receptor-mediated endocytosis; IV, endosomal/lysosomal escape; V, ATP-triggered Dox release in the cytosol; VI, accumulation of Dox in the nucleus. Adapted with permission from (Mo et al., 2014).

The concentration of ATP in tumor cells is extremely high due to the demand for rapid tumor growth, and, as such, can be used as an internal trigger for responsive nanomedicines. The two major mechanisms of ATP responsiveness are (I) competitive combination with the designed single stranded DNA (ssDNA) aptamers, and (II) structural degradation due to ATP consumption (Sun and Gu, 2016; Deng and Walther, 2020; Sameiyan et al., 2021). Gu’s group developed an ATP-responsive nanogel (Dox/NG) consisting of a DNA motif containing an ATP aptamer and its complementary strand with doxorubicin insertion, protamine, and a hyaluronic acid (HA)-crosslinked shell (Mo et al., 2014). In the presence of ATP, the ATP aptamer in Dox/NG competitively combined with ATP to form a stable tertiary structure and the loaded doxorubicin was released. When arriving at tumor tissue via the EPR effect and HA-mediated targeting, Dox/NG is degraded by HAase and releases the Dox-intercalated duplex. Subsequently, the Dox-intercalated duplex dissociates at a significantly higher level of ATP and releases dox to kill tumor cells (Figure 4). Gu’s group also induced graphene oxide (GO) aggregation through a combination of an ATP aptamer and two complementary single-stranded chains. This aggregation could be disrupted by the high concentration of ATP in tumor cells to release the loaded cargoes (Mo et al., 2015). Liu’s group developed an alginate-based hydrogel conjugated to the APT aptamer, which was hybridized with the immunoadjuvant CpG oligonucleotide. After intratumoral injection followed by chemotherapy or radiotherapy, alginate-based hydrogel can gradually release the immunoadjuvant CpG in a ATP-dependent manner to enhance the anti-tumor effect of immunogenic cell death (ICD) (Sun et al., 2021). Ramezani’s group developed a mesoporous silica nanosystem (dox@MSNs-Apts) modified with mucine-1 and ATP aptamers on the surface to form a Y-shaped DNA structure. As a gatekeeper, the ATP aptamer prevented the rapid release of dox from the pores of the MSNs. When encountering a high concentration of ATP after tumor cell endocytosis, ATP aptamers were competitively bonded with ATP, leading to the release of dox. Their results indicated that dox@MSNs-Apts showed greater cytotoxicity than the nanoparticles decorated with scrambled ATP aptamers (dox@MSNs-Apts scrambled) in C26 and MCF-7 cell lines (Bagheri et al., 2021). Willner’s group modified the metal-organic framework caged configurations (NMOFs) using the ATP aptamer for controlled drug release and the AS1411 aptamer for tumor targeting. Both aptamers and their complementary strands on the surface of NMOFs functioned as gatekeeper. Aptamer-decorated NMOFs release the loaded dye or drug in the presence of ATP (Chen et al., 2017a). Many studies have also indicated that ATP has high bonding affinity with Zn+, which can be used for detecting ATP. According to this phenomenon, nanoparticles with Zn+ in the structure might become unstable and degraded under ATP exposure. Chen’s group engineered self-assembled quantum dots (QDs)-phenolic nanoclusters (NCs) consisting of QDs, tannic acid (TA) and chemotherapeutics by metal-phenolic coordination. As they speculated, ATP could competitively bind to TA and dissolve the QD cluster (Song et al., 2018). Yang et al. also developed an ATP-responsive zeolitic imidazole gramework-90 nanoparticle according to a similar mechanism. Imidazole-2-carboxaldehyde, Zn^2+^, and protein self-assembled into nanoparticles in the solution. When ZIF-90/protein nanoparticles were exposed to ATP, their structure was degraded to release the loaded proteins (RNase and Cas9), thereby demonstrating the effectiveness of ZIF-90 nanoparticles. Additional biological processes that ATP is involved in also provide inspiration to develop ATP-responsive DDSs (Yang et al., 2019a). Yuan et al. used the cage-like protein GroEL to load chemotherapeutic dox, which could be activated by ATP molecules to release encapsulated dox. When incubated with ATP molecules, GroEL exhibited a rapid dox release profile *in vitro*, while *in vivo* anti-tumor experiments also indicated that this ATP-responsive delivery system could efficiently inhibit tumor growth (Yuan et al., 2018). The examples of ATP-responsive DDS of nanoparticles are summarized in Table 5.

**TABLE 5:** ATP-responsive DDS and examples.

Nanoparticles	Cargoes	Release Mechanism	Cell Lines and Animal Models	References
Hyaluronic acid nanogel	Dox	The structural change of duplex-to-aptamer induced by the formation of the ATP-aptamer complex	MDA-MB-231, MDA-MB-231 tumor mouse model	Mo et al. (2014)
DNA-graphene hybrid nanoaggregates	Dox	The dissociation of the aggregates induced by the formation of the ATP-aptamer complex	HeLa	Mo et al. (2015)
Alginate-based hydrogel	CpG oligonucleotide	The dissociation of ATP-Aptamer/CpG-cAptamer structure induced by the formation of the ATP-aptamer complex	CT26, CT26 tumor mouse model	Sun et al. (2021)
Mmesoporous silica nanoparticles	Dox	The dissociation of Y-shaped DNA structure induced by the formation of the ATP-aptamer complex	CHO, C26, MCF-7, C26 tumor mouse model	Bagheri et al. (2021)
Metal–Organic framework nanoparticles	Dox, Rhodamine 6G	The formation of the ATP-aptamer complex	MCF-10A, MDA-MB-231	Chen et al. (2017a)
Self-assembled quantum dots-phenolic nanoclusters	Dox	Structure disassembly induced by ATP competitively bonding with Zn^+^	HepG2, HepG2 tumor mouse model	Song et al. (2018)
Protein nanoparticles	Dox	ATP induced conformational change	MDA-MB-231, Panc-1, HPNE, 293T, Panc-1 tumor mouse model, MDA-MB-231 tumor mouse model	Yuan et al. (2018)

ATP is the major energy source in each living cell in our body, especially in some tissue with high metabolism. Although the level of ATP in tumor tissue is relatively higher than that in other normal tissues, nonspecific drug release is still the major obstacle for ATP-responsive DDSs. Exploring the response strategy with ATP concentration selectivity is an important research direction.

### 3.5 GSH-Responsive DDS

**FIGURE 5:**
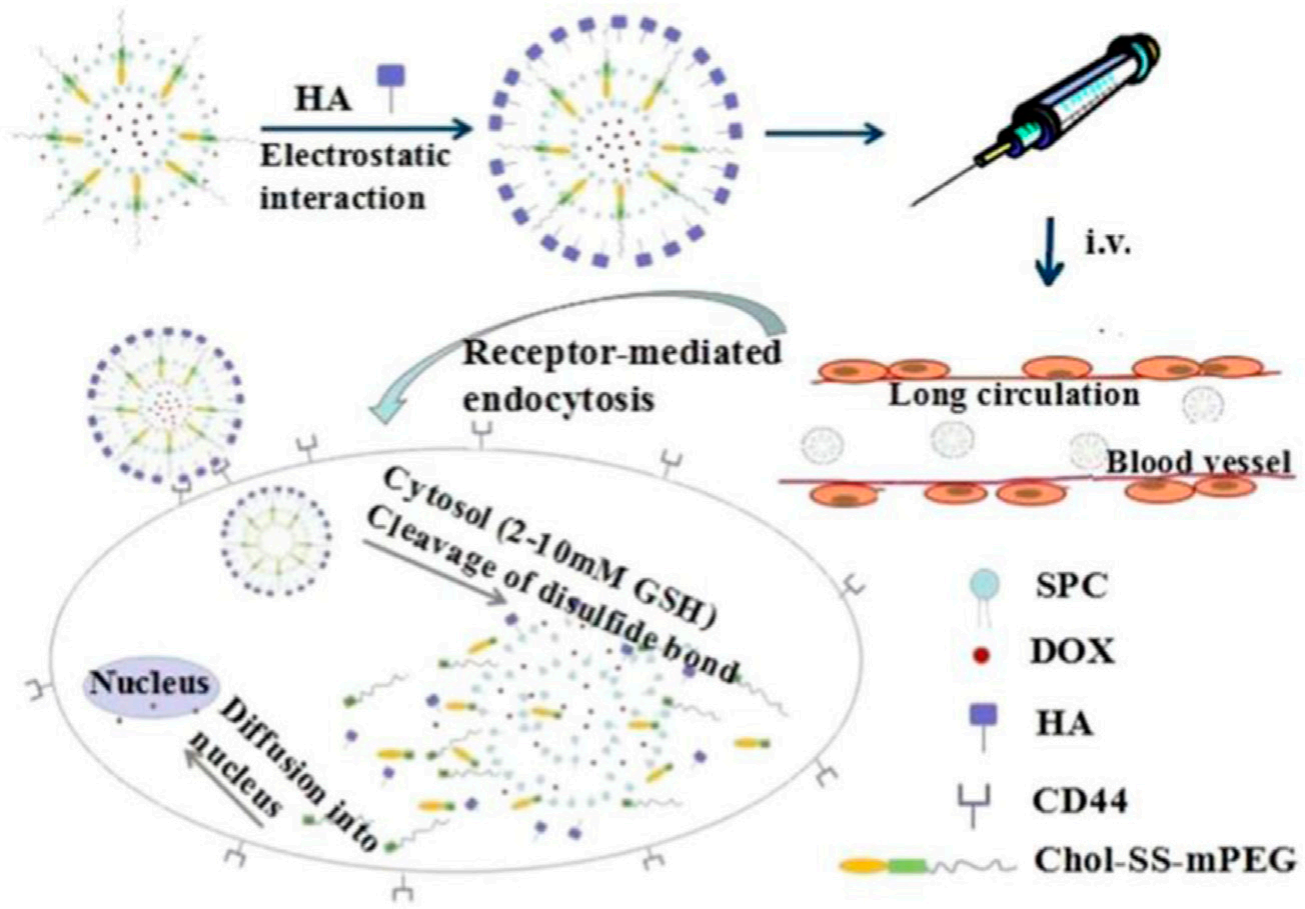
A Chol-SS-mPEG and HA dual-functionalized liposome (Chol-SS-mPEG/HA-L) designed for long circulation followed by receptor-mediated endocytosis and GSH-triggered cytoplasmic dox release. Adapted with permission from (Chi et al., 2017).

Compared to normal tissues, the concentration of GSH in tumor tissues is much higher (∼four times), while the concentration of extracellular GSH (∼2–10 μM) is approximately 1,000 times higher than that in intracellular (∼2–10 mM) compartments, which can be used as an effective internal stimulus for responsive drug release (Guo et al., 2018; Raza et al., 2018; Mollazadeh et al., 2021). Mahato’s group synthesized redox-sensitive mPEG-co-P(Asp)-g-DC-g-S-S-GEM polymer, in which the chemotherapeutics gemcitabine (GEM) were conjugated by a disulfide bond. They also synthesized another similar polymer mPEGco- P(Asp)-g-TEPA-g-DC to form a complex along with miR-519c, which could reverse hypoxia-induced drug resistance in pancreatic cancer. Upon incubation with l-glutathione, approximately 90% of GEM was rapidly released. With surface modification by the epidermal growth factor receptor targeting peptide GE11, the prepared nanoparticles could efficiently accumulate in tumor tissue and significantly inhibit tumor growth (Xin et al., 2020). Qiu et al. conjugated the anti-cancer drug camptothecin (CPT) on the surface of AgNPs via a GSH-responsive disulfide bond to realize controlled drug release under glutathione (GSH) conditions (Qiu et al., 2018). Liu’s group linked the functional siRNA onto the surface of mesoporous silica nanoparticles (MSNs) by disulfide linkage, termed MSNs-SS-siRNA@Dox. The surface linkage of siRNA also served as a gatekeeper to prevent the burst release of loaded small molecular drugs. The introduction of the disulfide bond into the block structure endows nanoparticles with GSP-responsive properties (Zhao et al., 2017). Wu’s group developed redox-sensitive and CD44-targeted liposomes, consisting of a novel detachable polyethylene glycol (PEG 2000) conjugated to cholesterol through a bio-reducible disulfide linker (Chol-SS-mPEG), with SPC, cholesterol, cationic lipid DOTAP, DOPE, and hyaluronic acid (HA) non-covalently coated on the surface (Chi et al., 2017). The chemotherapeutic dox was also loaded (Chol-SSmPEG/HA-L), and an on vitro release investigation indicated the prepared lipid nanoparticles showed a burst drug release of 60% in the presence of 10 mM glutathione (GSH) compared to non-redox sensitive liposomes. Chol-SSmPEG/HA-L exhibited the most effective tumor suppression ratio *in vivo* (Figure 5). Zhang’s group also developed a liposome-based nanosystem (PTX/siRNA/SS-L) incorporating a redox-sensitive cationic oligopeptide lipid, which not only had a proton sponge effect to improve lysosomes escape, but also induced the disruption of nanostructure in the presence of GSH. PTX/siRNA/SS-L showed a synergistic inhibitory effect on tumor growth and pulmonary metastasis in a 4T1 tumor animal model (Chen et al., 2017b). Jiang’s group reported hyaluronic acid (HA) nanogels, which were crosslinked by a disulfide linker, with chemotherapeutic dox encapsulated into the nanogels. The results revealed that HA nanogels exhibited a high *in vitro* and *in vivo* RHAMM-mediated cellular uptake and drug delivery, leading to effective inhibition of tumor growth and metastasis (Yang et al., 2017b). Materials containing intrinsic disulfide bonds can be used for constructing a GSH-responsive drug delivery system. Lei et al. used 4,4′-dithiobisbenzoic acid (4,4′-DTBA) containing disulfide bond as an organic ligand to prepare a metal-organic framework nanostructure (MOF-Zr (DTBA) loaded with curcumin (CCM). MOF-Zr (DTBA) displayed faster release behavior in the presence of GSH *in vitro* and enhanced tumor growth inhibition compared to free CCM. Similar to other internal responsive strategies, GSH-triggered systems can also be achieved by fabricating a gatekeeper outside the nanoparticles (Lei et al., 2018). Shi’s group developed a GSH-responsive dox-loaded nanographene oxide (NGO-SS-mPEG). The decorated PEG on the surface was conjugated by a disulfide bond and served as a gatekeeper to prevent the release of the Dox. NGO-SS-mPEG showed faster drug release upon intracellular GSH stimulation than in the absence of GSH (Wen et al., 2012). Wang’s group also coated EPG on the surface of mesoporous silica nanoparticles (MSNs) via a disulfide bond to realize controlled dye release. These PEG modifications can also improve the biocompatibility of nanomaterials (Wang et al., 2015). Except for PEG molecules, which serve as the gatekeeper, low-molecular-weight heparin (LMWH) can be used to decorate nanoparticles via disulfide bonds (Tian et al., 2016). In addition, GSH-responsive polymersomes are another important drug delivery system. Hubbell’s group synthesized block copolymers of the hydrophile poly (ethylene glycol) (PEG) and the hydrophobe poly (propylene sulfide) (PPS) with disulfide linkage to fabricate nanoparticles for intracellular drug delivery (Cerritelli et al., 2007). Koul’ group synthesized an amphiphilic triblock copolymer poly (polyethylene glycol methacrylate)-poly (caprolactone)-s-s-poly (caprolactone)-poly (polyethylene glycol methacrylate) (pPEGMA-PCL-ss-PCL-pPEGMA) with a disulfide linkage that self-assembled into polymersomes with a size of ∼150 nm. The GSH-responsive polymersomes showed ∼59% drug release in pH 5.5 in the presence of 10 mM GSH and ∼85% tumor regression as compared to free doxorubicin (∼42%) (Kumar et al., 2015). In addition to disulfide bonds, ditelluride bonds are also used to construct GSH-responsive nanocarriers. Sun’ group synthesized a folic acid (FA) modified PEGylated polycaprolactone copolymer via a ditelluride linkage and prepared the Dox-loaded micelles (F-TeNPDOX). F-TeNPDOX was able to release drugs in a GSH-dependent manner through the degradation of ditelluride bonds and promoted drug accumulation and enhanced growth inhibition in 4T1 tumor *in vivo* (Pang et al., 2020). Xiang’ group reported a ditelluride-bridged mesoporous organosilica nanoparticle (DTeMSN) decorated with polyethylene glycol-curcumin (PEG-CCM) on the surface to realize GSH-responsive drug release. In this work, ditelluride bond was utilized as a linkage to prepare MSNs for the first time. DTeMSNs@Dox@PEG-CCM gradually degraded under redox conditions and allowed sustained Dox release. They also compared the redox responsive behavior of Te-Te-bridged MSNs and Se-Se-bridged MSNs (Xia et al., 2022). Zhang’ group fabricated a novel ultrasensitive redox-responsive system using ditelluride bond as a responsive element for the controlled release of doxorubicin (DOX). In this work, the ditelluride group was introduced for the first time into water-soluble copolymers. The synthesized copolymer nanoparticles released Dox in a GSH-dependent manner and exhibited specific tumor targeting and antitumor activity (Wang et al., 2016). The examples of GSH-responsive DDS of nanoparticles are summarized in Table 6.

**TABLE 6:** GSH-responsive DDS and examples.

Nanoparticles	Cargoes	GSH-Responsive Efficiency (pH = 7.4)	Cell Lines and Animal Models	References
Polymer nanoparticles	Gemcitabine	∼90% in 10 mM GSH within 24 h	MIA PaCa-2, MIA PaCa-2R, HPDE, hPSCs, MIA PaCa-2R tumor mouse model	Xin et al. (2020)
Polymer prodrug/AgNPs hybrid nanoparticles	Camptothecin	∼90% in 10 mM GSH within 24 h	HeLa	Qiu et al. (2018)
Mesoporous silica nanoparticles	siRNA and doxorubicin	∼78.8% in 5 mM GSH within 24 h	MCF-7, MCF-7 tumor mouse model	Zhao et al. (2017)
Liposome nanoparticles	Doxorubicin	>60% in 10 mM GSH in the first 4 h	LO2, MG63, MG63 tumor mouse model	Chi et al. (2017)
Oligopeptide liposomes	Paclitaxel, siRNA	>80% in 10 mM GSH within 36 h	4T1, 4T1 tumor mouse model	Chi et al. (2017)
Hyaluronic Acid Nanogels	Doxorubicin	About 69% in 10 mM GSH within 120 h	LNCaP, H22, A549 and NIH3T3; H22 and LNCaP tumor mouse models	Yang et al. (2017b)
Metal−Organic Framework Nanocarriers	Curcumin	>80% in 10 mM GSH within 24 h	HeLa, MDA-MB-231, HeLa tumor mouse model	Lei et al. (2018)
Mesoporous silica	Rhodamine B	60% in 10 mM GSH within 24 h	MCF-7	Wang et al. (2015)
Polymersomes	Doxorubicin	∼40% in 10 mM GSH within 24 h	BT474, MCF-7, L929, Ehrlich’s ascites tumor mouse model	Cerritelli et al. (2007)
Mesoporous organosilica nanoparticle	Doxorubicin	∼80% in 5 mM GSH within 24 h	HeLa, HeLa tumor mouse model	Xia et al. (2022)

In general, high level of GSH is detected in the intracellular region, determining GSH-responsive DDS is just used for the delivery of drugs that work within tumor cells. Biological barriers including tumor blood vessels and cell membranes significantly hamper the efficacy of cellular internalization. Moreover, the concentration of GSH is just four times higher in tumor cells than that in normal cells, which makes the design of GSH-sensitive DDS difficult.

### 3.6 Hypoxia-Responsive DDS

**FIGURE 6:**
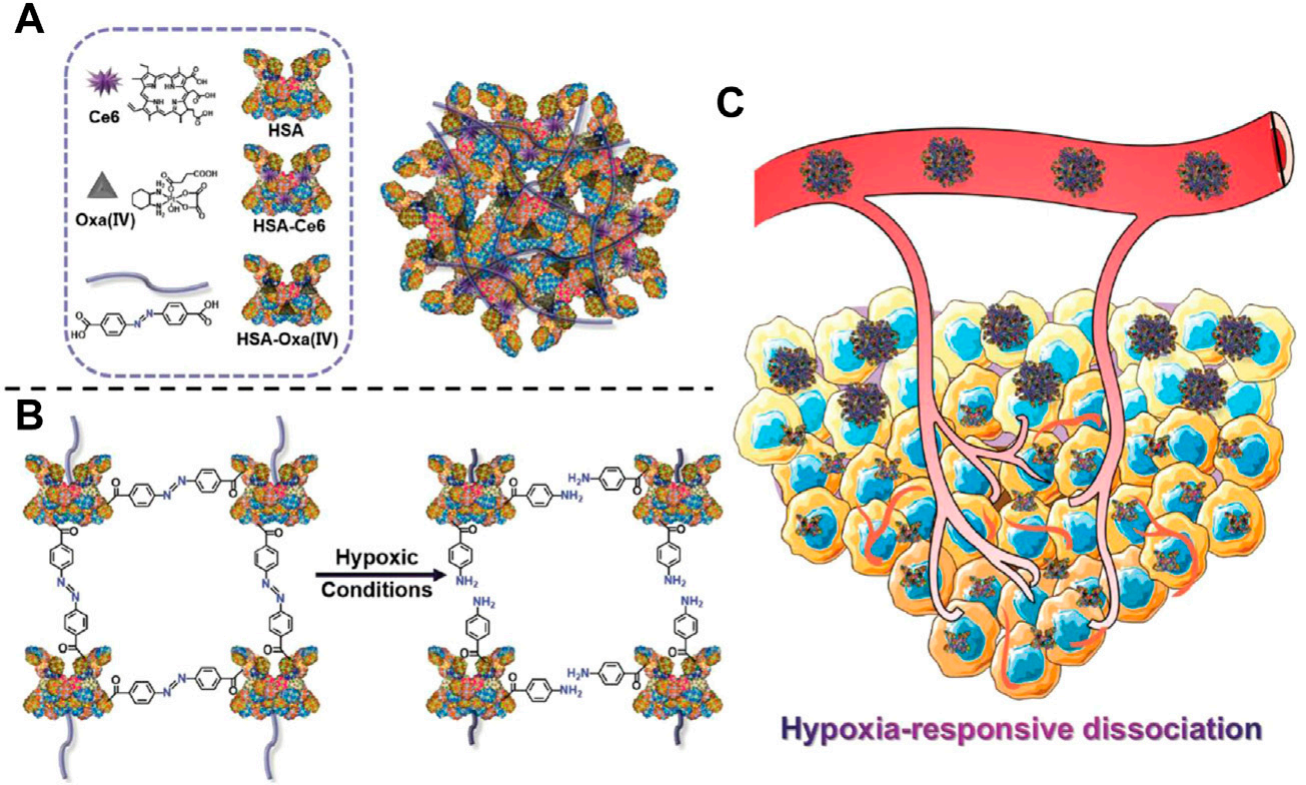
Schematic illustration of HCHOA and functional mechanism. **(A)** Schematic illustration of HCHOA and its components. **(B)** Scheme indicating the hypoxia-responsive dissociation of HCHOA. **(C)** Scheme showing that, in the microenvironment of tumor hypoxia, hypoxia-responsive HCHOA could disintegrate into individual HSA-based (Human Serum Albumin) complexes with improved tumor penetrating ability. Adapted with permission from (Yang et al., 2019b).

The contradiction between the unsatisfied demand for nutrients and oxygen in the tumor region and the excessive tumor growth and metabolic strains leads to the formation of structurally defective and irregular tumor vasculature, which induces the hypoxic tumor microenvironment. Hypoxia is an important hallmark of tumors that is highly associated with angiogenesis, invasiveness, metastasis, and drug resistance, and can also be taken advantage of to design responsive DDSs (Thambi et al., 2016; Li et al., 2020e). Generally, three types of moieties can be used to realize hypoxia responsiveness, including azo linkers, nitrobenzyl alcohol, and nitroimidazoles (Zhou et al., 2020). Yang et al. prepared a hypoxia-responsive human serum albumin (HSA)-based nanosystem (HCHOA) by conjugating chlorin e6 (Ce6)-conjugated HSA (HC) and oxaliplatin prodrug-conjugated HSA (HO) via an azobenzene linker (Figure 6). In the circulation, HCHOA was stable and maintained a size of 100–150 nm, which could accumulate in tumor tissue through EPR effects. When arriving at tumor tissue and being exposed to the hypoxic tumor microenvironment, HCHOA quickly dissociates into HC and HO therapeutic nanoparticles with a diameter smaller than 10 nm, which showed enhanced tumor penetration capability. Their results demonstrated that HCHOA could efficiently target tumor tissue and presented a synergistic effect of photodynamic therapy and chemotherapy combination (Yang et al., 2019b). Torchilin’s group developed a hypoxia-responsive nanocarrier (PAPD) consisting of polyethyleneglycol 2000, polyethyleneimine (PEI), and 1,2-dioleyl-sn-glycero-3-phosphoethanolamine (DOPE), in which the PEG and PEI groups were linked by azobenzene. In the hypoxic tumor microenvironment, DOPE could detach the PEG group and expose the positively charged PEI to enhance cellular endocytosis and further transfection efficiency (Perche et al., 2014). Zhang’s group conjugated hydrophobic combretastatin A-4 (CA4) with hydrophilic irinotecan (IR) via an azobenzene (AZO) bond with hypoxia sensitivity to self-assemble into nanoparticles (IR–AZO–CA4/CP NPs) in solution. IR–AZO–CA4/CP NPs showed hypoxia-responsive properties and enhanced tumor penetration (Zhang et al., 2016). In addition to the azobenzene linker, the nitrobenzyl group is also widely used to synthesize hypoxia-responsive nanocarriers. Indeed, Xiao’s group prepared a hypoxia-responsive amphiphilic polymer by conjugating a hydrophobic small molecule, 4-nitrobenzyl (3-azidopropyl) carbamate onto the side chains of an mPEG-PPLG copolymer. The amphiphilic polymer self-assembled into nanoparticles loaded with dox (PPGN@dox). PPGN@dox presented a hypoxia-sensitive drug release behavior *in vitro* and increased antitumor activity *in vivo* via optimizing the pharmacokinetic properties. Nanoparticles with a positively charged surface exhibit enhanced tumor penetration capability; thus, nanoparticles with a negative charge in the circulation and positive charge in the tissue exhibit great potential to enhance anti-tumor activity (Zhang et al., 2020). Shi’s group developed a hypoxia-responsive nanocarrier, composed of a poly (caprolactone) core and a mixed shell of PEG and 4-nitrobenzyl chloroformate (NBCF)-modified polylysine (PLL). This nanocarrier can gradually increase the positive surface charge by responding to the hypoxic tumor microenvironment and penetrating deep into tumor tissues (Zhen et al., 2019). Nitroimidazole is another important hypoxia-responsive motif. Park’s group conjugated the carboxymethyl dextran with a hydrophobically modified 2-nitroimidazole derivative to establish hypoxia-responsive nanoparticles (HR-NPs), which simultaneously encapsulated dox. HR-NPs showed a hypoxia-dependent drug release profile *in vitro* and selectively accumulated in hypoxic tumor tissues to realize significant anti-tumor activity *in vivo* (Thambi et al., 2014). Zhu’s group incorporated nitroimidazole derivatives into the liposome structure to form hypoxia-responsive liposomal drug delivery system, termed dox-HR-LPs after encapsulation with dox. They demonstrated that dox-HR-LPs had high sensitivity to hypoxia and released dox in an oxygen-dependent manner. *In vivo* anti-tumor experiments also indicated that dox-HR-LPs exhibited enhanced therapeutic efficacy in both cell line-derived and patient-derived xenograft models (Li et al., 2019b). The typical examples of Hypoxia -responsive DDS are summarized in Table 7.

**TABLE 7:** Hypoxia -responsive DDS and examples.

Moieties	Nanoparticles	Cargoes	Cell Lines and Animal Models	References
Azobenzene	Albumin-Based Nanosystem	**Ce6,** oxaliplatin	4T1, 4T1 tumor mouse model	Yang et al. (2019b)
Azobenzene	Polymer nanoparticles	siRNA	HeLa, A2780, NCI-ADR-RES, B16F10 tumor mouse model	Perche et al. (2014)
Azobenzene	Drug–drug conjugated nanoparticles	Combretastatin A-4 hydrophilic irinotecan cyclopamine	MCF7	Zhang et al. (2016)
Nitrobenzyl	Polymer nanoparticles	Doxorubicin	4T1, 4T1 tumor mouse model	Zhang et al. (2020)
Nitrobenzyl	Polymer nanoparticles	Doxorubicin	MDA-MB-231, MDA-MB-231 tumor mouse model	Zhen et al. (2019)
Nitroimidazole	Polymeric nanoparticles	Doxorubicin	SCC7, SCC7 tumor mouse model	Thambi et al. (2014)
Nitroimidazole	Liposomes nanoparticles	Doxorubicin	RM-1, FaDu, RM-1 tumor mouse model, patient-derived xenograft (PDX) lung cancer model	Wang et al. (2016)

Hypoxia is a universal feature in different tumor types. However, hypoxia level in different area within the same tumor tissue is quite different. The hypoxia level in the central region of the tumor is usually much more severe than that in the peripheral region. Therefore, how to efficiently deliver hypoxia-responsive DDSs into the central region is an important problem. In addition, the relationship between the response rate and hypoxia level is also worth investigating.

## 4 Conclusion and Perspectives

Over the past decades, great advances have been achieved in the development of bio-responsive DDS and their applications in cancer therapy in preclinical studies. A rationally designed bio-responsive DDSs can overcome the difficulties encountered by many traditional chemotherapeutic drugs and provide a new way to treat tumors, which have important clinical application prospects, e.g., by improving the efficacy of traditional chemotherapy, controlling drug release, enhancing tumor specificity, improving pharmacokinetics/pharmacodynamics, reducing toxic side effects, and decreasing drug resistance. Despite the great efforts made in scientific publications, few bio-responsive DDSs have been successfully applied in clinical trials. Here, we summarize the recent progress of bio-responsive DDSs based on their specific characteristics in the tumor microenvironment (acidosis, overexpression of special enzymes, hypoxia and high levels of ROS, GSH and ATP), with the aim of providing a strong guideline for the design of bio-responsive DDS and their applications. Among these specific characteristics, most of them are universal in different tumor types, e.g., acidosis, hypoxia, ROS, GSH and ATP. Only the overexpressed enzymes are different and need to be selected according to tumor type.

In addition to the frequently applied DDS above-mentioned, multi-stimuli-responsive DDSs have been developed to overcome the dilemmas that single stimulus-responsive DDS face in terms of specificity and programmed release of different drugs, e.g., pH and enzyme combination, pH and ATP combination, pH and ROS combination, and pH and GSH combination (Jiang et al., 2019; Wang et al., 2020b; Du et al., 2020; Meng et al., 2020). To overcome the multiple biological barriers that hinder the drug delivery, Wang’ group described pH/cathepsin B hierarchical-responsive nanoconjugates (HRNs) which presented different states in different physiological environments. HRNs with an original size of 40 nm in the circulation were beneficial to tumor accumulation and dissociated into polymer conjugates with a size of 5 nm in an acidic tumor environment to facilitate deep tumor penetration. After being endocytosed into the lysosomes, the conjugates are cleaved by cathepsin B to release chemotherapeutics DTX into cytoplasm and inhibit cell proliferation. Moreover, this system can induce *in vivo* antitumor immune responses and is suitable for combination with immune therapy, e.g., *a*-PD-1 (programmed cell death 1) therapy. The results showed that HRNs in combination with *a*-PD-1 exhibited synergistic antitumor efficacy (Du et al., 2020). By endowing one nanocarrier with multi-stimuli-responsive properties, programmed drug delivery can be achieved. Liu’s group developed a pH/ROS/MMP-2 triple-responsive drug delivery system using the designed poly (ethylene glycol)-peptide-poly (*ω*-pentadecalactone-co-N-methyldiethyleneamine-co-3,3′-thiodipropionate) (PEG-M-PPMT) copolymer for enhanced tumor accumulation and deep penetration. In the presence of MMP2 in tumor the microenvironment, PEG-M-PPMT nanoparticles can partially shed PEG corona to form smaller particles and penetrate deep into the tumor tissue. Under the acidic endosomal pH conditions and high intracellular ROS levels, PEG-M-PPMT nanoparticles substantially swelled and released loaded drugs. All these properties of PEG-M-PPMT lead to an extraordinary combined chemo/photodynamic therapy (Shu et al., 2021). Zeolitic imidazole frameworks (ZIFs) have natural acid and ATP response features. Jiang et al. decorated ZIF90 nanoparticles using the Y1 receptor ligand [Asn6, Pro34]-NPY (AP) on the surface for tumor targeting. Combining targeted delivery and dual responsive release of DOX significantly improves the therapeutic efficacy of ZIF90 nanoparticles in MDA-MB-231 tumor bearing mouse (Jiang et al., 2019). Sun’ group integrated a pH-sensitive polymer octadecylamine-poly (aspartate-1-(3-aminopropyl) imidazole) (OA-P(Asp-API)), a ROS-generation agent and *ß*-Lapachone (Lap) into one nanosystem modified with RGD (iRGD)-modified ROS-responsive paclitaxel (PTX)-prodrug. The OA-P(Asp-API) in formed RLPA-NPs easily protonates in the endosome’s acidic environment and escape from endosome. The released lap produced ROS which triggered the release of PTX from PTX-prodrug to kill tumor cells (Li et al., 2020f). Chen’s group synthesized a GSH and pH dual sensitive polypeptide-DEX conjugate (L–SS–DEX) to self-assemble into nanoparticles. L–SS–DEX showed enhanced acid-triggered and GSH- triggered drug release *in vitro* and superior antitumor activity with an approximately 86% tumor suppression rate in comparison with 49% in the free DEX group (Ma et al., 2020). However, these combination strategies also increase the complexity of DDS and bring difficulties for their clinical application. Besides, external stimuli (magnetic fields, illumination and temperature) can also be used in combination with biological stimuli (Fang et al., 2021). Additionally, the low specificity of single stimuli, sluggish response speed, and poor biocompatibility of applied nanomaterials require further optimization. With the emergence of new technologies, such as nanomanufacturing technology and click chemistry, intelligent responsive DDSs with higher drug-forming potentials are expected to be developed. Although responsive DDSs based on the tumor microenvironment have been extensively explored, their clinical application still faces many challenges. First, there is a large heterogeneity among cancer patients, and the tumor microenvironment varies significantly in different types of tumors or different tumor growth stages (Pe’er et al., 2021). Therefore, preclinical models that better reflect tumor heterogeneity for evaluating bio-responsive DDSs should be established. Moreover, the complex interactions between the tumor extracellular matrix and tumor cells have not been fully clarified. Second, the *in vivo* degradability and biocompatibility of the materials have not been comprehensively verified in vivo experiments. The novel and multi-functional detecting systems need to be developed to monitor the pharmacokinetic process, toxicology-related characteristics, *in vivo* bio-distribution, and metabolism of drug delivery systems, which will greatly promote the development of intelligent drug delivery systems. Third, the biosafety including organ toxicity and immunogenicity of selected materials should be evaluated in depth. Fourth, how to precisely control the various physicochemical characteristics of complex drug delivery systems, e.g., size, shape, surface charge, stability, and functionalization, remains an important aspect for clinical translation. The structural and/or mechanistic complexity of multi-stimuli-responsive DDSs often restricts their clinical translation. A possible solution for this dilemma is the synthesis of motifs with two or more responsive properties to reduce their complexity. In addition, nanoparticles often have to travel the complex biological barriers to arrive at tumor cells, limiting the efficacy of nanomedicines (Niu et al., 2018; Feng et al., 2019). It is universally acknowledged that nanoparticles with a size of less than 50 nm have a short circulating half-life and can easily penetrate deep into tumor tissue but not tend to be trapped. In contrast, nanoparticles with sizes of 50–200 nm exhibit long circulating half-life and effective tumor accumulation and retention. One of the promising strategies to solve this issue is the development of multistage nanoparticles by the rational design of bio-responsive DDSs. Wang’s group reported a pH-responsive clustered nanoparticle to realize size changes from ∼100 nm in the circulation to ∼5 nm in the tumor tissue. By doing this, they achieved long blood circulation for effective tumor accumulation and deep tumor penetration. The functional process of this well-designed multistage nanosystem was demonstrated by penetration experiments using *in vitro* 3D tumor multicellular spheroids and an *in vivo* BxPC-3 xenograft tumor model (Li et al., 2016). Ren’s group synthesized an ultrafine single-chain tadpole polymer consisting of an intrachain crosslinked globule and a pH-sensitive linear polymer chain to fabricate polymer nanoparticles in solution, simultaneously encapsulating chemotherapeutic paclitaxel (PTX/MTAs). PTX/MTAs showed a size-changeable property in different pH condition and achieve deep tissue penetration in tumors (Song et al., 2019). He’s group developed a smart size-switchable nanoplatform (DGL/DOX@PP) by conjugating small dendrigraft poly-l-lysine (DGL) to poly (ethylene glycol)–poly (caprolactone) micelles via a matrix metalloproteinase 2 (MMP-2)-responsive peptide. When DGL/DOX@PP with a size of 100 nm accumulated in tumor tissue by the EPR effect and encountered overexpressed MMP2 in tumor microenvironment, small dox-loaded nanoparticles (∼30 nm) were rapidly released and penetrated into the deep area of tumor tissue (Cun et al., 2018). Many other stimulus-sensitive size shrinkable drug delivery systems have also been developed to overcome the tumor stromal barrier (Sun et al., 2015). Recent studies have indicated that immune cells in the tumor microenvironment also influence drug delivery. Weissleder’s group reported that tumor-associated macrophages (TAM) in the tumor microenvironment serve as a local drug depot that stores drug-loaded nanoparticles and are gradually released into to tumor cells (Miller et al., 2015). TAM depletion decreased the tumor accumulation of nanomedicine, indicating that we can optimize drug delivery efficacy by regulating immune cells in the tumor microenvironment.
